# Twitter Activists’ Argumentation Through Subdiscussions: Theory, Method and Illustration of the Controversy Surrounding Sustainable Fashion

**DOI:** 10.1007/s10503-022-09579-1

**Published:** 2022-09-06

**Authors:** Sara Greco

**Affiliations:** Institute of Argumentation, Linguistics and Semiotics, USI-Università della Svizzera italiana, Via G. Buffi 13, 6900 Lugano, Switzerland

**Keywords:** Subdiscussions, Material starting points, SUBDIMA, Twitter, Locus from effect to cause, Polylogue

## Abstract

“Why are millions of dollars worth of orders being left unpaid?”. With tweets like this questioning brands’ policies, activists advocating for sustainable fashion re-discuss material starting points that are assumed by fashion brands, who argue that they are sustainable because they care about their workers’ conditions. This paper argues that activists use tweets to open *subdiscussions on material starting points* to engage citizens and consumers, re-discussing factual *data* that brands take for granted, such as the fact that they provide fair conditions for their garment workers. Activists justify their opening of subdiscussions, often through an argumentative pattern that includes an argument based on the *locus from effects to cause*. They argue that if there are negative effects, the brand cannot claim to care about the conditions of its workers. In discussing how subdiscussions are used by fashion activists, this paper also introduces a conceptualization of Twitter argumentation as a discussion that is not isolated, but is part of a polylogical argumentation that takes place in different venues. For this reason, the argumentation used in tweets is reconstructed as a response to a fashion brand’s communication campaigns around sustainability, which extend beyond the confines of Twitter. As an empirical illustration, this paper is based on the campaign targeting fashion retailer Primark; the dataset includes the brand’s website as well as activists’ tweets.

## Introduction: Positioning of the Problem, Research Questions

This paper introduces the hypothesis that Twitter activists make use of tweets to open argumentative subdiscussions to promote their cause, using the example of activists who advocate for environmental and social sustainability in the fashion industry. I argue that activists’ tweets may be opening *subdiscussions on material starting points* (henceforth: SUBDIMAs), calling into question material premises (facts, values, or knowledge propositions) that are taken for granted by fashion brands when they declare themselves sustainable in their communication campaigns; campaigns which are expressed in argumentative texts located outside Twitter. In order to understand activists’ tweets as opening SUBDIMAs, I assume the concept of argumentative *polylogue* (Lewiński and Aakhus 2014), and in particular the idea that polylogues happen in different “places (mostly in the sense of *venues*)” (Aakhus and Lewiński 2017, p. 195). On this basis, I claim that fashion activists’ tweets should not be considered as isolated productions; they should be reconstructed as reactions to argumentations published elsewhere. Consequently, the argumentative nature of tweets should be evaluated taking into account that the argumentative discussion (van Eemeren and Grootendorst 2004) might exceed the confines of the Twitter platform.

Hence, this paper addresses the following research questions:

RQ1: Do activists’ tweets open SUBDIMAs? What is their function in relation to activists’ goals? Is the opening of SUBDIMAs justified? Are there argumentative patterns that recur in these justifications?
RQ2: In terms of empirical methodology, how should we analyze polylogical argumentation that takes place in different venues? What are the consequences for the reconstruction of Twitter argumentation?

While the first group of research questions addresses content, the second group regards the establishment of a *method* for analyzing broad polylogical argumentative discussions. Thus, this paper contributes to argumentation studies in two different ways. Firstly, it considers SUBDIMAs in the context of fashion activists’ campaigns on Twitter, hence illuminating an argumentative strategy (the opening of SUBDIMAs) and context (social media campaigns on sustainable fashion) that have not been investigated before. Secondly, going beyond studies that look at argumentative discussions on the Twitter platform, this paper introduces the methodological suggestion of interpreting Twitter messages within broader argumentative discussions taking place in different venues.

This paper will proceed as follows. In Sect. 2.1, I will explain the theoretical framework related to polylogical argumentation and the notion of different venues, moving on to Twitter argumentation in the controversy surrounding sustainable fashion (2.2). Section 2.3 will focus on the concept of subdiscussion, illustrating the theoretical basis of this work. Section 3 will contain methodological considerations, which are particularly important in this paper because, beyond illustrating the method of the present analysis, they show how to study argumentation in polylogue empirically. Section 4 will present the findings of the empirical analysis, while Sect. 5 will discuss these in relation to the research questions. Finally, Sect. 6 will draw conclusions and outline further research steps.

## The Concept of Subdiscussions in Activists’ Campaigns

### Consequences of the Notion of *Multiple Venues* on the Analysis of Argumentative Discussions in Polylogical Public Controversies

The focus of this paper is on the public controversy surrounding sustainable fashion, which can be interpreted as a polylogical argumentative discussion. Lewiński and Aakhus (2014) introduce the notion of polylogue to address “not simply a discussion between multiple participants, but rather multiple different argumentative parties defending their distinct positions” (2017, p. 181). These authors argue that “disagreement expansion” in polylogical discussions “not only occurs over positions and players but it also expands over place” (Aakhus and Lewiński 2017, p. 182). Hence, they introduce the idea that places or venues should be included in the analysis of polylogues:

“By venue we aim to capture the dual sense of where and when actors come together without limiting encounters to a geographical location and to highlight how actors come together, the instrumentation, in a variety of ways (e.g. interpersonal, institutional, mass mediated; orally, textually)” (Aakhus and Lewiński 2017, p. 185).

Earlier, Kerbrat-Orecchioni (2004, pp. 3–4) noted that polylogue is a gradable concept. She considered a *trilogue* (e.g. a three-party discussion at the dinner table) as the “minimal form taken by polylogues” (p. 4). Public controversies are more extreme cases of polylogue, because a variety of actors participate in them, and the polylogue is “prolonged in its duration and contains an element of polarization or even conflict” (Greco and De Cock 2021, p. 58). The concept of different venues is particularly important for public controversies because it redefines the boundaries of argumentative discussions, considering that it is possible for someone to respond to others who are arguing in a different venue. However, Aakhus and Lewiński (2017) do not delve into the empirical consequences of considering different venues for argumentation analysis. In this paper, I will show that considering different venues changes how an analyst should interpret what happens in each individual venue. Specifically, it may be that a message posted on social media should not be read as an isolated argumentative event but as the response to some other argumentative text published in a different venue. Hence, the boundaries of argumentative discussions and of individual venues do not coincide.

In the specific case considered in this paper, activists open subdiscussions by responding to claims and arguments made by brands in other (different) venues, such as their websites. Subdiscussions so far have been analyzed mostly within discussions that take place in one venue (e.g. a family discussion, as in Schär 2021) whereas this paper considers SUBDIMAs in a *cross-venue* perspective. As a consequence, empirically, if one wants to study the argumentation potential of activists’ SUBDIMAs, one needs to reconstruct the contributions previously made by fashion brands, which appeared in different venues, and consider these different texts as one argumentative discussion.

The analyst’s perspective on Twitter argumentation may radically change if one considers Twitter not as an isolated discussion venue but as part of a broader network of venues within a polylogue. Recently, Twitter argumentation has been the subject of growing interest, both in argument mining research (for an overview, see Schäfer and Stede 2021) and in argumentation studies in general (Mohammed 2019; Goodwin 2020; Elliott-Maksymowicz et al. 2021; Greco et al. 2021; Foderaro and Lorentzen 2022) as well as in discourse analysis (Roginsky and De Cock 2015). However, to the best of my knowledge, these studies consider argumentation within the confines of Twitter itself, either in individual tweets or in conversational threads. Schäfer and Stede (2021, p. 46) note that “given its fast-paced and sometimes superficial nature it is reasonable to question if argumentation actually takes place on Twitter”: I claim that in order to evaluate the argumentative nature of individual tweets in fashion activists’ campaigns, one should consider them in a cross-venue perspective. In the following section, I will detail this hypothesis in relation to the controversy surrounding sustainable fashion.

### The Public Controversy Surrounding Sustainable Fashion: Twitter Activism and Argumentation

The public controversy surrounding sustainable fashion has been ongoing since the nineties, but more so since 2000, when fast fashion brands changed their business model, introducing weekly collection updates (Wallinger 2015).

Various actors are involved in this controversy, including fashion brands, policymakers, activists, other institutions (e.g. museums) and citizens, who are concerned both in terms of their civic engagement and as consumers of fashion (Greco and De Cock 2021). Sustainable fashion activism as a form of political discourse originated before the advent of social media (Balsiger 2014) but in recent years it has been amplified by the opportunities social media affords. For example, multiple organizations were set up in the aftermath of the Rana Plaza accident, which killed over 1000 garment workers in Indonesia, leaving more than two thousand others wounded (De Castro 2021). Activist organizations regularly question the social and environmental sustainability of the fashion industry; some activists focus exclusively on fashion (e.g. Fashion Revolution) while others address various domains (e.g. Greenpeace, whose Detox campaign targeting fashion was analyzed in Brambilla 2019). These concentrate on raising citizens-consumers’^1^ awareness of the fashion industry’s potential lack of transparency. Although this paper focuses on activist organizations, it is important to note that social media activists include other contributors in the controversy surrounding sustainable fashion, such as small brands, who use social media to amplify their voice (Tuite 2018) and promote their collections and products as sustainable items. Moreover, we also find individual citizens and consumers communicating about their experience with sustainable fashion, often gathering around rallying hashtags (Karamalak and Cantoni 2021) launched by activist organizations, such as #whomademyclothes.

### Opening Subdiscussions on Material Starting Points in Twitter Argumentation

At this point, a discussion on the theoretical concepts that underpin this paper is needed. In Sect. 2.3.1, I will discuss the concept of argumentation taken from pragma-dialectics, its relation to polylogue and controversy, and the connected notions of subdiscussions on material starting points and argumentative patterns. To specify types of subdiscussions that target specific material starting points, Sect. 2.3.2 will introduce the Argumentum Model of Topics.

#### Pragma-Dialectics as a Model for the Reconstruction of Discussion and Subdiscussion

Pragma-dialectics sees argumentation as a dialogic process to resolve a difference of opinion “on the merits”, i.e. “by means of argumentative discourse” (van Eemeren 2018, p. 34). As van Eemeren (2018, p. 34) puts it,

“The abstract notion of a critical discussion has been introduced in pragma-dialectics to represent the theoretical ideal of an argumentative discourse optimally instrumental in putting the acceptability of the standpoint at issue in the difference of opinion to the test”.

I consider that interpreting argumentation as a dialogical process of a reasonable critical discussion is a valid philosophical ideal, which is applicable to polylogical argumentation. Moreover, the concrete concepts and instruments considered in pragma-dialectics are also important for analyzing polylogues.^2^

The model of a critical discussion sets out four stages, corresponding to “the different phases an argumentative discourse must pass through for resolving a difference of opinion on the merits” (van Eemeren 2018, p. 36). In the confrontation stage, “it becomes clear that there is a standpoint that meets with real or projected doubt or contradiction” (ibid.). In the opening stage, which is central in this paper, the “procedural and content-related material commitments” are identified (ibid). This stage can often be implicit. In the argumentation stage (van Eemeren 2018, p. 37), the protagonist and antagonist (or, in a polylogical situation, the various participants) defend their standpoints and provide supporting arguments. In the concluding stage, it is determined “whether the protagonist’s standpoint has been properly defended against the critical responses of the antagonist” (ibid.).

These stages may be present in discussions and subdiscussions alike (van Eemeren and Grootendorst 2004, p. 147).^3^ This paper specifically considers subdiscussions that redefine material starting points in the opening stage (SUBDIMAs). In SUBDIMAs, the discussants may “allow for a subdiscussion to be conducted in which it is determined whether the proposition on which agreement was first lacking can be accepted in the second instance” (van Eemeren and Grootendorst 2004, p. 147). By definition, SUBDIMAs emerge as subordinate to a main discussion. Opening a SUBDIMA thus means that a material starting point that was taken for granted is “called out” (van Eemeren et al. 1993, pp. 95ff; Musi and Aakhus 2018; Jackson 2019). The called-out proposition, “made problematic within the discourse, functions as a ‘virtual standpoint’ in need of defense” (van Eemeren et al. 1993, p. 95).

As in any argumentative discussion, SUBDIMAs may be developed to a greater or lesser extent. Some remain at the confrontation stage only; others may include an argumentation stage or even reach a concluding stage. When activists’ SUBDIMAs include an argumentation stage, it is possible to identify *argumentative patterns*.^4^ These are characterized by “a constellation of argumentative moves in which, in order to deal with a particular kind of difference of opinion, in defense of a particular type of standpoint a particular argument scheme or combination of argument schemes is used in a particular kind of argumentation structure” (van Eemeren 2017, pp. 19–20).

#### Types of Subdiscussions on Material Starting Points

Because this paper deals with subdiscussions on material starting points, it is worth taking a closer look at the different types of starting points in argumentative inference, which might become virtual standpoints in the opening of SUBDIMAs. To this aim, this paper adopts the Argumentum Model of Topics (AMT, Rigotti and Greco 2019). In the AMT, the *inferential configuration* of each individual argumentation supporting a standpoint includes two interconnected components: a “procedural-inferential” component based on *loci* as inferential sources of arguments, which is abstract and decontextualized; and a “material-contextual” component based on intersubjective agreement between the arguers (p. xiii). Within the material-contextual component, *endoxa* and *data* constitute two different types of starting point. *Endoxa* include general knowledge and values that play the role of major premises, while *data* are factual premises that play the role of minor premises. Integrating this distinction, we observe that activists’ SUBDIMAS on *endoxa* or *data* target different levels of argumentative inference.

Although the analysis in this paper is centered on verbal content, some basic considerations of the role of (often multimodal) images will be included. Drawing on Groarke (2019) and Tseronis (2020), we assume that multimodal elements contribute to inference and look to identify the contribution of pictures in SUBDIMAs that are justified by arguments within the identified argumentative patterns.

## Empirical Illustration: Dataset and Method of Analysis

### Activists Versus Primark: A specific Example of Fashion Activists’ Campaigns

Given its purpose of introducing a new conceptualization of SUBDIMAs and a new method of analysis, this paper focuses on an in-depth analysis of a case study. The selected case relates to Irish fashion retailer Primark, owned by Associated British Foods, which can be ascribed to the category of fast fashion (Dach and Allmendiger 2014). In the 2021 Fashion Transparency Index published by Fashion Revolution, Primark’s overall score was in the range between 31 and 40%, which is the medium category of the classification.^5^ However, its score was lower for traceability (within the 21–30% range, at 23%) and for the so-called “spotlight issues”, which in 2021 unsurprisingly focused on problems related to workers’ wages and the cancellation of orders due to the pandemic. On 1 November 2020, Clean Clothes declared their intention to launch a campaign on these issues, and mentioned Primark as one of the four “companies that have most impact”.^6^ In June 2021, Clean Clothes published a report significantly entitled “Breaking point: Wage theft, violence and excessive workloads are pushing garment workers to breaking point during the pandemic” (Clean Clothes 2021), based on an analysis of interviews with garment workers producing items for Nike, Primark and H&M. They reported that “All three companies have returned to making considerable profits in past months” (p. 5) while they “are clearly not doing enough to protect workers from the financial impact of the Covid-19 crisis” (p. 3). The selected campaign against Primark (started by Clean Clothes but then taken up by other activist organizations) can be seen as representative of a typical *modus operandi*: activists repeatedly target one particular brand to demand more transparency and sustainability.

### Criteria Governing the Collection of Data

In view of the polylogical controversy considered in this paper, the dataset is composed of two main data genres. The first is the text of the Primark website that promotes the “Primark Cares” campaign (www.primark.com/en-gb/primark-cares) while the second is a dataset of tweets by activists.

For the first, I collected data on the “Primark Cares” sustainability campaign, as this might be considered the main target of activists. Details of the “Primark Cares” campaign are described on their website, the content of which was collected manually on 5 July 2021. Other documents published by Primark owner Associated British Foods (e.g. CSR reports) are available,^7^ but these ultimately direct the reader to the “Primark Cares” website for further information. It therefore seemed sensible to concentrate on the content of the website itself.

For the second data genre, I collected a Twitter dataset from three activist organizations whose purpose is to advocate for sustainable fashion. The Twitter dataset was collected on 1 July 2021 using the NCapture extension of the NVIVO software, which was made available at the author’s institution (USI—Università della Svizzera italiana). Using a method inspired by Orminski et al. (2021, p. 7), three influential organizations which could be defined as activists for sustainable fashion were identified, namely Fashion Revolution, Clean Clothes and Labour Behind the Label. First, we knew that these organizations often tweeted about sustainable fashion from previous research (Greco et al. 2021). Second, as Orminski et al. (2021, p. 7) observe, “According to the literature, the number of followers and self-created tweets indicate opinion leadership” and all three NGOs have a significant number of followers and tweets on sustainable fashion. All the collected tweets are in English. NVIVO collects data from Twitter respecting the company’s terms and conditions. After careful reflection on the ethical implications of social media research (see for example Giglietto et al. 2012; Ahmed et al. 2017), the decision was made to anonymize references to private individuals even though they have public accounts on Twitter. Mentions of Primark and of the activist organizations under consideration have been maintained because arguably their social media posts are intended to be public.

**Fig. 1:**
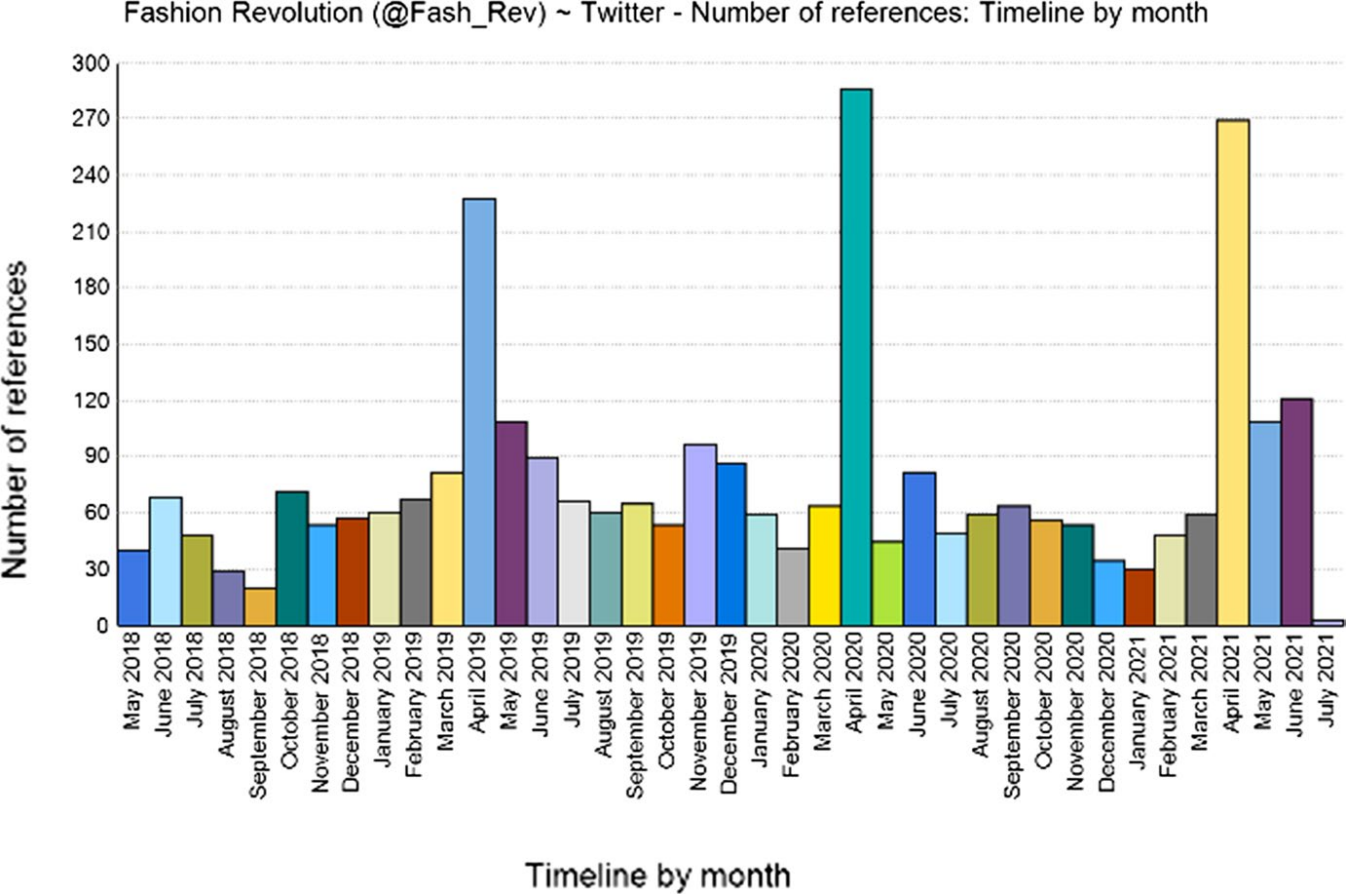
Tweets by fashion revolution by month (full dataset). Chart taken from NVIVO

I will now briefly describe the three selected organizations in greater detail. Fashion Revolution (@Fash_Rev) was created in the aftermath of the Rana Plaza accident in 2013, and as of 1 July 2021 (the day of the data collection) had 56,954 followers on Twitter. Every year, they publish the Fashion Transparency Index and engage social media users in their Fashion Revolution week, which takes place in April to coincide with the anniversary of the Rana Plaza accident. Clean Clothes Campaign (@cleanclothes) was founded in the early nineties in The Netherlands (Sluiter 2009).^8^ As of 1 July 2021, it had 24,205 followers on Twitter. It focuses on “Amplifying worker voices in the garment and sportswear industry”.^9^ During the Covid-19 pandemic, Clean Clothes has focused on campaigns relating to “wage theft”, asking brands to pay their workers (see the hashtag #PayYourWorkers), as several ordered were cancelled during lockdowns, including orders that had already been prepared. Labour Behind the Label (@labourlabel) declares on its Twitter profile that they “represent Clean Clothes in the UK”. As an organization, it is less international but still has a significant number of followers on Twitter (13,831 followers as of 1 July 2021). The production of tweets by the three NGOs is not constant over time, with Fashion Revolution, for example, publishing more tweets in April, i.e. the Fashion Revolution Week month (Fig. 1). However, despite these variations in frequency, the three NGOs identified regularly tweet or retweet content relating to sustainable fashion.

### Data Curation and Analysis

The content of the “Primark Cares” homepage was copied and analyzed manually. The full Twitter dataset included 9485 tweets (including retweets, thus partially overlapping) from Fashion Revolution, Clean Clothes Campaign, and Labour Behind The Label. From this, I selected tweets relating to Primark and the “Primark Cares” campaign (Total = 149 tweets, including retweets). As a second limitation, I only considered tweets that received 10 or more retweets. This choice was made to guarantee that activists’ potential opening of SUBDIMAs was successfully perceived as a new discussion, indicated through a minimum number of retweets, which were assumed as a rough estimation of resonance. The final dataset included 81 tweets.

As for the procedure of analysis, the “Primark Cares” website was first analyzed, using the AMT model (see Sect. 4.1) to reconstruct its argumentative structure, the main argument schemes (loci) and their implicit premises. The Twitter dataset was annotated manually, using Microsoft Excel. The following categories were annotated: type of standpoint (see Table 2 below), presence of a SUBDIMA (yes/no), type of material starting point targeted in the case of a SUBDIMA (*endoxon/datum*), presence of an argumentation stage in the SUBDIMA (yes/no), *locus*, use of questions to open SUBDIMAs, presence of an image (yes/no/image consisting solely of wording), and function of the image in the inference.

## Findings

In this section, I will first present the argumentative reconstruction of the “Primark Cares” campaign (4.1), before then moving on to the reconstruction of activists’ subdiscussions (4.2).

### The “Primark Cares” Campaign: Argumentative Analysis

As a first step, the argumentation contained in the homepage of the “Primark Cares” campaign was reconstructed. The relevant text that was analyzed is reported in “Appendix 1”. In the following reconstruction, number 1 indicates the standpoint, while the subsequent numbers indicate arguments and subordinative arguments (cf. van Eemeren 2018).^10^

1. Primark cares about sustainability.
  1.1 The welfare of the people who make products for Primark matters to us.
    1.1.1 Whether they’re making t-shirts in Bangladesh, or socks in Turkey or jewelry in India, we expect wages to be fair and working conditions safe.
  1.2 We strive to minimize impact on the planet wherever we can.
  1.3 Every factory that manufactures products for Primark must commit to meeting internationally recognized standards.

The reconstruction presents a series of arguments based on the *locus from definition* (Rigotti and Greco 2019), namely 1.1, 1.2, and 1.3, which support standpoint 1 independently. The use of definitions is perhaps not surprising, considering that the aim of the “Primark Cares” website is to show that this brand is sustainable. Definitional arguments were also found in previous research on sustainable fashion (Greco and De Cock 2021). The present analysis concentrates on the relation between standpoint 1 and argument 1.1 and the subordinative argumentation connecting 1.1.1 to 1.1, because these two inferences are the ones particularly targeted by the activists’ campaigns, as will be shown in Sect. 4.2.

Argument 1.1, which supports standpoint 1, details why it is reasonable to say that “Primark Cares” about sustainability; in other words, what is defined is the meaning of “caring for sustainability”. The subordinative argument 1.1.1, supporting 1.1, is also based on a *locus from definition*, this time justifying why it can be said that Primark cares for “the people who make products for Primark”. The inferential rule (*maxim*, see Rigotti and Greco 2019) that underpins the *locus from definition* is as follows: “if and only if x has the characteristic of a given species, then x belongs to that species” (see Schär 2017). Adopting this maxim as a basic inferential rule for the *locus from definition*, the AMT makes reference to the Aristotelian concept of *specific difference*: the definition identifies the specific difference needed for a certain entity in a genus to be ascribed to a species (Rigotti and Greco 2019, p. 45). In arguments 1.1 and 1.1.1 (Table 1), Primark uses an argument based on the *locus from definition* to support the standpoint that the brand “cares about sustainability”: the specific difference “caring for the welfare of the people who make products” defines its belonging to the species of “brands that are sustainable”. Subsequently, the brand uses subordinative argumentation to support the fact that it cares about its workers, on the basis of the specific difference of “expecting wages to be fair and working conditions safe” being the specific difference that positions Primark within the species of “brands that care about their workers”.

**Table 1:** AMT reconstruction of the material starting points in arguments 1.1 and 1.1.1

Argument	Endoxon	Datum
1.1 (supporting 1)	Being sustainable means caring for the welfare of the people who make products	We care about the welfare of the people who make products
1.1.1 (supporting 1.1.)	Caring for the welfare of the people who make products means that (whether they’re making t-shirts in Bangladesh, or socks in Turkey or jewelry in India) means that one should expect wages to be fair and working conditions safe	(Whether people make t-shirts in Bangladesh, or socks in Turkey or jewelry in India), we expect wages to be fair and working conditions safe

Table 1 synthetizes an overview of the AMT analysis, which details the material-contextual premises associated with the maxim of the *locus from definition* in the two inferential passages being considered. In Sect. 4.2, we will see how activists target these premises.

### Activists Opening SUBDIMAs: Findings

#### Types of Standpoint in Activists’ Tweets

If one takes into account that activists are responding to the “Primark Cares” campaign, findings show that the entire dataset (with the exception of 2 tweets) is composed of tweets that contain argumentation. The structure of the argumentative tweets relates to three different types of standpoint, which can be formulated as in Table 2. We refer here to the distinction between *evaluative* and *prescriptive* standpoints discussed in van Eemeren (2017, p. 17).

**Table 2:** Types of standpoints in tweets

	Type of standpoint	Example	Frequency
a.	An *evaluative* standpoint, normally implicit: “Primark does not really care about its workers”	(1) “These 29 young Burmese women garment workers were just fired at @Primark’s Amber Stone factory in Myanmar for organizing a union. Issuing BLM statements while firing the workers who make your clothes for speaking up during a pandemic?! Unbelievable Primark” (LabourLabel Retweet, 16 February 2021)	17/81
b.	A *prescriptive* standpoint, which can be both implicit and explicit: “Primark should perform a certain action” (e.g. ‘pay your workers’)	(2) “Workers are literally going hungry because brands are failing to take responsibility for their supply chains. What are @Primark and @Mango doing to ensure that the workers who made their clothes before the pandemic started are receiving their full wages now? #PayYourWorkers” (LabourLabel, Retweet 15 February 2021)	43/81
c.	A *prescriptive* standpoint, normally explicit, that targets its readers: “The readers of this tweet should perform a certain action” (e.g. ‘Tell Primark to #PayUp now!’)	(3) “Did you know that workers are literally going hungry during the pandemic because they're not paid their full wages? A recent research showed that 67% out of 400 interviewed workers and their families had been forced to skip meals in the past months. Tell @Primark #PayYourWorkers https://t.co/CYkATv4ZYA” (CleanClothes, Tweet, 31 January 2021)	19/81

The three types of standpoint illustrated in Table 2 are interrelated. In fact, in (b) and (c), the standpoint (a) is used as an argument to justify the pragmatic standpoint. In (c), consumers/citizens are invited to ask brands to do something (hence, standpoint (b) is included); in turn, this is often justified with (a).

The predominance of (b), i.e. a prescriptive standpoint asserting that Primark should do something different, might suggest that this brand is the primary addressee of the campaign. Primark is also often mentioned explicitly using the opportunity offered by Twitter as a platform: @Primark. In some cases, the tweets target Primark individually, while in other cases Primark is addressed together with other companies, such as Topshop, Inditex (Zara), and H&M, but also a luxury brand like Armani, and others. However, Primark is not the real addressee, at least not on Twitter, as activists tend not to expect a direct response from the brand via Twitter (see the discussion in Balabanova and Palmieri 2020); it is rather an “unaddressed ratified reader” (Palmieri and Mazzali-Lurati 2016, p. 480), who in this case is relevant because activists want them to know that the campaign has been launched and could lead consumers to become more aware and more critical.

#### Activists’ SUBDIMAs and their argumentative pattern

The evaluative statement “Primark does not really care about their workers”, which might be a standpoint (as in a.) or an argument (as in b. and c.) can be considered as the opening of a SUBDIMA, as it re-discusses the brand’s *data* in arguments 1.1 or 1.1.1, i.e., that Primark cares about its workers by paying them fair salaries and making their working conditions safe. 74 out of the 81 tweets in the selected corpus can be read as opening a SUBDIMA; all these SUBDIMAs target a *datum* (and not an *endoxon*).

Among the 74 tweets that can be read as opening SUBDIMAs, none includes a concluding stage, because, as mentioned in Sect. 4.2.1, brands tend not to respond on Twitter. However, in 66 of the 74 cases, these SUBDIMAs include an argumentation stage, which is often composed of a single couple standpoint + argument or a standpoint + two (subordinative) arguments.^11^

Analysis of the dataset revealed different variants of a frequent argumentation pattern being used to support the statements “Primark does not really care about their workers” or “Primark does not provide fair working conditions”, which contains an argument based on a *locus* connecting *effects to cause*. As Rigotti and Greco (2019, p. 96) argue, drawing on the medieval notion of *habitudo* (which translates as *relation*), each *locus,* understood as a source of inference, is a relation between two poles. Hence, each *locus* can be read in two different directions (from A to B or, conversely, from B to A). In this case, the direction of reading of the cause-effect relation is: *from effects to cause*. Hence, the corresponding maxim in this specific case is: “If the effects do not correspond to a certain cause, that cause is not active”. Activists use this maxim to argue against Primark’s declaration of sustainability. In fact, they prove that there are effects experienced by garment workers working for Primark or its subcontractors, which are incompatible with “caring about workers” or “expecting wages to be fair and working conditions safe”. These effects are not described as a whole; each tweet tends to consider *one* concrete example, e.g. workers who are not paid, or are not supported in their legitimate strike activity, etc.

Depending on the type of standpoint (Table 2), the argumentative pattern occurs in different variants; but the *locus from effects to cause* is present in 56 out of the 66 cases of SUBDIMAs that contain argumentation. The remaining ten cases contain arguments from definition, cause to effect, and authority.

**Fig. 2:**
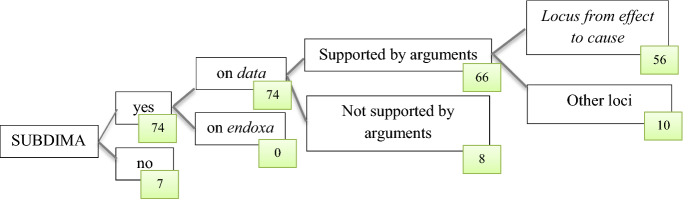
Summary of the results

Figure 2 presents a synoptic overview of the findings derived from the dataset used in this paper.

Although the analysis of SUBDIMAs in this paper is based on their verbal component, it is important to add a note on the function of the many images found in the corpus (64 of the 74 tweets containing SUBDIMAs are accompanied by some form of image, either multimodal or consisting entirely of wording). Roughly half of the images (31 out of 64) can be said to have a function that accompanies the main argumentative pattern, centered on the *locus from effects to cause*. The 31 images related to this *locus*, in fact, either show the effects of the brand’s policies (e.g. garment workers suffering) or portray garment workers protesting in different countries. In both cases, these images corroborate the inference expressed verbally, showing through visual support the existence of the negative effects mentioned. Beyond this notable use of images to complement the *locus from effects to cause* by visually emphasizing effects, other functions of images can be found: some contribute to argumentation in different ways (e.g. showing the standpoint, or adding further arguments through visual or verbal elements), while only 15 out of the 64 images only represent the situation generically, without a comprehensible link to argumentative inference.

## Discussion

In this section, the answers to this paper’s two main groups of research questions will be discussed.

*RQ1*: *Do activists’ tweets open subdiscussions? What is their function in relation to activists’ goals? Is the opening of subdiscussions justified? Are there argumentative patterns that recur in these justifications?*

The majority of the tweets included in the final dataset are argumentative^12^ and, if considered in relation to the “Primark Cares” campaign, they do open SUBDIMAs. As noted in Sect. 4.2.1, Primark is an unaddressed ratified reader of these discussions. We might say that there is a mixed argumentative discussion between Primark and the activists, while the main argumentative discussion is with citizens-consumers, with activists trying to raise their awareness of the (un)sustainability of the fashion industry. Gregory (2010, p. 90) describes this goal as: “getting target publics to think about something and trying to promote a level of understanding”. Balsiger (2014) observes that the “success” of activists’ campaigns depends on their capacity to raise new points in the agendas of consumers, retailers and even brands. Brands are monitored over time by fashion activists to check for progress; in general, we observe that activists’ campaigns tend to be successful, if we consider that “consumers interested about sustainability [in fashion] represent today quite a relevant ‘niche’” (Rinaldi 2019, p. 19).^13^

The opening of SUBDIMAs in our context is an important move per se, because it constitutes the act of questioning a brand’s (implicit) starting point. Therefore, the success of SUBDIMAs should not be measured by their reaching a concluding stage, also because activists often do not expect brands to respond to their campaigns on Twitter, but on their establishing discussion on propositions taken for granted by brands. It is striking that in 28 cases, the opening of a SUBDIMA is linguistically formulated as a question, which emphasizes the fact that the activists’ goal is to shift the discussion onto a different issue. In two cases in the dataset, questions include a reformulation of the hashtag #Primarkcares, turning it into a question: #Primarkcares?, thus explicitly questioning the brand’s standpoint. In other words, activists reclaim the right to discuss in the public arena the value of the sustainability that brands lay claim to and, thanks to the use of Twitter as a venue, they avoid the risk of communication being unidirectional from brands to citizens and consumers. As Balsiger (2014, p. 1) puts it, “activists put the question of the social and environmental conditions in the garment industry on the agendas of clothing retailers and in the mind of consumers. (…) And they directly targeted and publicly exposed firms to make them acknowledge the problem and change their policies and practices”. Metaphorically, we could say that activists use SUBDIMAs as an “argumentative wedge”, driving it into the discussion between brands and consumers to re-discuss what brands take for granted.

Two other aspects deserve some discussion. Firstly, the findings of this paper show that the majority of SUBDIMAs include an argumentation stage. Findings show different variants of argumentative standpoints (pragmatic or evaluative, Sect. 4.2.2) which are frequently supported by a *locus from effects to cause* (Sect. 4.2.2). As stated in Sect. 4.2.2, this *locus* often selects one specific example of effect to show that the cause “caring for people” is not actually present in Primark’s practice. The choice of a single negative effect as a counterexample might be linked to the argumentative strength of counterexamples. Sinnott-Armstrong and Fogelin (2015, p. 334) observe that “One common way to refute a premise is by showing that it is false by producing a counterexample. Counterexamples are typically aimed at universal claims”. In this sense, one counterexample is sufficient to show that the “Primark Cares” claim is not completely true. Additionally, the frequent inclusion of a single counterexample selected from among many possible may be partly explained through the restriction of the number of characters in a Twitter message, which imposes constraints on the type of argumentation possible on this platform (see for example Schäfer and Stede 2021, p. 46). It has been noted before that Twitter argumentation is often enthymematic due to the limitations of the number of characters (Elliott-Maksymowicz et al. 2021).

Secondly, it is striking that all SUBDIMAs found in the empirical dataset concern *data* rather than *endoxa*. Re-discussing the *datum* of brands’ sustainability is in line with previous research, which has shown that sustainability per se is a fuzzy value (Greco and De Cock 2021). Ours is certainly not an isolated case of *data*-targeting SUBDIMAs: campaigns against other brands are based on the same strategy. Between the end of 2021 and the beginning of 2022, for example, Clean Clothes Campaign launched a campaign targeting the lingerie brand Victoria’s Secret. Playing on the term “secret”, activists accuse this brand of their real and “dirty” secret being that some of their workers have not been paid.^14^

One might wonder, however, if targeting *data* is the only strategy of fashion activists’ SUBDIMAs. Indeed, it seems that in other cases, *endoxa* are targeted. Take, for example, the Boycott Black Friday campaigns, which are launched every year around the time of the Black Friday commercial initiative (at the end of November), often using the hashtag #BoycottBlackFriday. During Black Friday, brands often argue that their products are worth buying (standpoint) because there are discounts on them (argument). A preliminary analysis of some of the tweets used in those campaigns seems to indicate that SUBDIMAs in these tweets often target not the *datum* but the *endoxon* itself, which can be formulated as “it is worth rushing out and buying a lot of new products on Black Friday when they are discounted”. Examples of *endoxa*-targeting SUBDIMAs assert that shopping as an activity is not in itself worthwhile, as in the following case: “The climate crisis is driven by overconsumption. I will be boycotting Black Friday. Please join me! Here are some ideas of what you could do instead on Friday and Saturday—let’s fill twitter with the positive alternatives! #BoycottBlackFriday (25 November 2021). Some assert that there are more important activities than shopping thus, again, going against the *endoxon* of Black Friday sales: “Happy Thanksgiving! Now stop checking social media and spend time with family and friends, no matter how much the corporations beg you to spend hard-earned money in stores today and tomorrow! #Thanksgiving #HappyThanksgiving #BlackFridayBlackout #BoycottBlackFriday #BuyNothingDay (25 November 2021). Further research would be needed to understand what type of activists’ campaigns are related to *endoxa* and the argument patterns in this case, and whether *data*-targeting and *endoxa*-targeting campaigns are related to different types of hashtags.

*RQ2*: *In terms of empirical methodology, how should we analyze polylogical argumentation that takes place in different venues? What are the consequences for the reconstruction of Twitter argumentation?*

With regard to RQ2, this paper has not only illustrated how fashion activists open SUBDIMAS to target fashion brands; it has also demonstrated in practice a method for the reconstruction of public controversies by empirically reconstructing argumentation that takes place in different venues (in the case considered in this paper, a website and Twitter). This paper proposes a new way of looking at Twitter’s argumentation, considering that the content of tweets may be interpreted, at least in some cases, as responses to arguments that have been made elsewhere. In a sense, this is a practical application of the Bakhtinian concept of *addressivity*: “Every word is directed towards an *answer* and cannot escape the profound influence of the answering word that it anticipates” (Bakhtin 1981, p. 279, emphasis in the original). In argumentative terms, this means that, at a theoretical level, in public controversies, analysts should be careful about where to set the boundaries of the discussions. At an empirical level, as argued previously by Greco and De Cock (2021), this means that analysis of public controversies will often include a composite dataset that encompasses different types of empirical data.

More specifically, the method and annotation scheme developed by this paper can be used for other activists’ campaigns. Tools such as INCePTION or UAM CT can be used to assist the analysis of larger datasets, helping to automatically find correlations in large datasets and across different activists’ campaigns.

## Conclusion

This paper has contributed to an understanding of the argumentative public controversy surrounding sustainable fashion through analyzing activists’ Twitter argumentation targeting the fashion retailer Primark. The analysis of a dataset created from the “Primark Cares” website devoted to sustainable fashion, and tweets from three activist organizations (Clean Clothes Campaign, Labour Behind the Label and Fashion Revolution) has shown that activists use tweets to open subdiscussions on the material starting points (in particular *data*) used by Primark in its claims concerning sustainability. Through this analysis, this paper has proposed a method for analyzing polylogical argumentation that takes place in different venues, considering that the reconstruction of Twitter argumentation should take into account the fact that the boundaries of an argumentative discussion may be broader than the boundaries of the platform. In this sense, this paper has made a contribution to argumentation studies across multiple aspects: a better understanding of the function of opening SUBDIMAs in activists’ argumentation, a contribution to clarifying the issues in the controversy that surrounds sustainable fashion as a subfield of environmental and political argumentation, and, no less importantly, a methodological contribution to interpreting Twitter argumentation in the context of polylogues.

This paper is part of a broader research project that analyzes argumentation in the controversy that surrounds sustainable fashion. Because this paper introduced a theoretical and methodological understanding of how SUBDIMAs can be used by activists, the empirical illustrative case focused on one campaign. In future, as mentioned in Sect. 5, the same type of analysis could be extended to other activists’ campaigns around sustainable fashion, to verify the extent to which their use of SUBDIMAs is similar. In particular, as the findings in this paper revealed that SUBDIMAs target *data*, it would be interesting to compare them with other campaigns in which activists arguably target *endoxa*, such as the campaigns concerning Black Friday (see the discussion in Sect. 5). Finally, as many fashion brands (Karamalak et al. 2021) and fashion activists also use Instagram, the research could broaden the dataset to include Instagram, taking into account the different opportunities the platforms afford (e.g. the necessary presence of images on Instagram or the different number of characters allowed).

Some aspects, which have not been foregrounded in this paper, will be interesting to explore as future research avenues. Firstly, given the frequent use of images in the dataset, one could focus on the analysis of their role in Twitter (and potentially Instagram), connecting multimodal argumentation to the inferential structure of arguments (Groarke 2019; Tseronis 2020). Secondly, the frequent presence of interrogative structures to open SUBDIMAs and the pragmatic and argumentative functions of questions (in text and in hashtags) could constitute an important aspect for a linguistic-based argumentative analysis. Again at the linguistic level, it would be interesting to look more closely at the nouns and phrases used to characterize brands’ garment workers, exploring *characterization frames* (Mercuri, in preparation): these range from the term “garment workers” to the more empowering “people who make our products” used by the brand. Finally, although this paper does not focus on the argumentative function of hashtags, it was briefly observed that hashtags expressed some of the standpoints (#BoycottBlackFriday) or issues of the SUBDIMAs (#PrimarkCares?). Hashtags can also express arguments (Greco and De Cock 2021). Further research could investigate the argumentative role played by hashtags as indicators of propositions at issue, standpoints, or arguments, developing existing classifications of the functions of hashtags (Zappavigna 2018, pp. 30–33). Moreover, the possible relationship between types of hashtags and *data*-targeting or *endoxa*-targeting SUBDIMAs could also be explored.

## Appendix 1

### Appendix 1 contains a citation of the contents of the “Primark Cares” Hom﻿epage as Retrieved on 5 July 2021^15^

#### “People and Production

The welfare of the people who make products for Primark matters to us. Whether they’re making t-shirts in Bangladesh, or socks in Turkey or jewellery in India, we expect wages to be fair and working conditions safe. Primark does not own factories. In fact, 98% of the factories making products for Primark also manufacture for other brands. We require every supplier and factory to commit to meet internationally recognised standards. Read about how we work with suppliers and factories to check whether these standards are being met.

(Read more-link).

#### Planet

The lifecycle of a Primark product involves many stages which can affect the planet. We strive to minimise this impact wherever we can. Whether it’s the cotton that goes into our t-shirts, the dyes used by factories or the way our products are transported and sold in-store. Find out about the steps we are taking to reduce our environmental footprint.

(Read more-link).

#### Setting High Standards

Every factory that manufactures products for Primark must commit to meeting internationally recognised standards before we place an order. We do not own factories and are very selective about who we work with. Once on our approved factory list, it’s the job of our Ethical Trade and Environmental Sustainability Team, a group of more than 100 experts based in key sourcing countries, to monitor compliance. In this section you will find information on the Primark Code of Conduct, the programmes and policies we have in place to positively impact standards within the garment and textile industry, and our annual statements on the Modern Slavery Act.

(Read more-link).

#### Global Sourcing Map

Primark does not own any factories and is selective about the suppliers with whom we work. Every factory which manufactures product for Primark has to commit to meeting internationally recognised standards, before the first order is placed and throughout the time they work with us.

(Read more-link)”.

## Footnotes

1 The terms *citizens* and *consumers* refer to the population in general. For brands, individuals are potential consumers. Activists address individuals both as consumers (to increase their awareness of the consequences of their shopping) and as citizens (to increase their awareness of the environmental and social consequences of the fashion industry).

2 Aakhus and Lewiński (2017, p. 182) observe that: “Applying the dyadic dialectical method is adequate, even necessary for some localized episodes of argumentative exchanges where two parties clash”. Even more so, it is important to have empirical analytical concepts, such as *subdiscussion* and *opening stage*, which are not contradicted by a polylogical view.

3 In argumentation studies, we find terms such as “metadiscussion” (van Eemeren et al. 1993, p 27; van Laar 2003, p. 201), as well as discussions on “meta-issues” (Dascal 1989/2003, p. 281), and “metadialogue” (Krabbe 2003; van Laar 2003). Though they do not always exactly coincide, the affixes “sub” and “meta” in these different accounts arguably point to similar phenomena: they indicate a discussion that is “inserted” or “embedded” (Krabbe 2003) within another “main” discussion.

4 Argumentative patterns in this case can be identified in relation to digital activists’ campaigns as an activity type. A complete discussion of this is beyond the scope of this paper but needs to be the subject of further research. I am indebted to José Alfonso Lomelí Hernández for this observation.

5 In particular, “Brands scoring between 31 and 40% are typically disclosing their first-tier manufacturers as well as detailed information about their policies, procedures, social and environmental goals, governance, supplier assessment and remediation processes. These brands are also more likely to be disclosing partial information on a few of the Spotlight Issues such as carbon emissions, gender equality, sustainable sourcing and materials and energy use” (Fashion Revolution 2021, p. 35).

6 See https://cleanclothes.org/news/2020/global-campaign-confronts-hm-primark-and-nike-with-unpaid-workers-voices (last visited: March 2022).

7 See https://www.abf.co.uk/responsibility (last visited: February 2022).

8 According to their website, they were founded “in 1989 as Schone Kleren Campagne” (https://cleanclothes.org/about, last visited: February 2022).

9 See https://cleanclothes.org/ (last visited: February 2022).

10 Except for argument 1.1, which is the one targeted by activists, I have not included subordinative argumentation in this reconstruction.

11 In many cases, tweets include links to external documents or other sources, which can contain further argumentation. However, the present analysis only includes the argumentation within the tweet itself.

12 This result differs from previous analyses on other datasets regarding sustainable fashion (Greco and De Cock 2021; Greco et al. 2021). The reason for the difference possibly lies in the fact that in previous datasets, we collected data about a generic hashtag (e.g. #sustainablefashion), around which different actors including brands and individuals gather, rather than on a specific activist campaign.

13 In the case discussed in this paper, the success of the campaign is confirmed by activists themselves. At some point, Labour Behind the Label tweets: “You did it! After months of campaigning, @Primark has committed to paying in full for their orders. Whether you signed our petition or called Primark out on social media- thanks for making them #PayUp! (…)”.

14 See also the website https://www.victoriasdirtysecret.co/ (last visited: March 2022), which, at the time of writing this paper, clearly juxtaposed the words of Victoria’s Secret’s CEO, quoted as saying “I have a bold ambition that Victoria’s Secret should be the world’s biggest and best advocate for women.”, and the words of a crying garment worker, who was quoted as asking “Why don't you start by paying us?”. This campaign is also conducted on social media; it targets *data*.
